# Prevalence of Hydatidosis in Cattle Slaughtered at Bishoftu Municipal Abattoir, Ethiopia, and Assessment of Its Economic Loss and Community Awareness

**DOI:** 10.1155/vmi/6938807

**Published:** 2024-11-22

**Authors:** Tesfaye Bekele, Nigate Fentaw, Ayichew Teshale, Solomon Mosu

**Affiliations:** Department of Veterinary Medicine, School of Veterinary Medicine, Wolaita Sodo University, Sodo, Ethiopia

**Keywords:** Bishoftu municipal abattoir, cattle, community awareness, economic loss, hydatid cyst and prevalence

## Abstract

**Background:** Hydatid disease is a zoonotic disease caused by the intermediate stage of dog tapeworm *Echinococcus granulosus*.

**Objectives:** The study investigated the prevalence of hydatid cysts and the risk factors in cattle slaughtered at the Bishoftu municipal abattoir. It also analyzed the economic loss and community's awareness of this matter.

**Animals**: 480 cattle brought for slaughtering were included in the study.

**Methods:** A cross-sectional study was conducted from December 2022 to April 2023 in Bishoftu municipal abattoir involving antemortem examination of cattle and postmortem hydatid cyst investigation, assessment of associated economic loss, and community awareness survey. Animals were selected systematically and cysts were investigated using standard meat inspection procedures.

**Results:** 10.4% of the slaughtered cattle were found positive for hydatid cysts, with no statistically significant association with the considered risk factors. From 85 collected cysts, the lungs accounted for 55.3%, the liver for 29.4%, and the remaining 15.3% were from the spleen, kidney, and heart. About 47.1% of the cysts were fertile and 52.9% were infertile, of which, 37.5% were nonviable. In a survey of 100 individuals, 33.0% were unaware of hydatid disease, and 61.0% were unaware that the disease is zoonotic. Annual economic loss due to the case was estimated at 5,444,564.4 ($97,224.4).

**Conclusion:** This study identified hydatid disease as a significant issue in cattle in Bishoftu, indicating the need for preventative and control measurements. Strategies such as deworming, controlling street dogs, establishing standardized abattoirs, and raising public awareness should be applied to alleviate the problem in Bishoftu and nationwide.

## 1. Introduction

Ethiopia has a large livestock population and is often said to be the largest in Africa. In the country, there are approximately 70 million cattle, 42 million sheep, 52 million goats, and 8 million camels [1]. Ethiopia produces livestock primarily through two systems: the sedentary mixed crop–livestock production system, which is mostly used in the highlands, and the agropastoral or nomadic pastoral production system in lowland areas [2]. The nation produces beef using the commercial feedlot system, small-scale peri-urban fattening, backyard fattening under the mixed crop–animal system, and the pastoral/agropastoral livestock production system [3]. In addition, more than 30% of agricultural laborers work in the livestock industry. The nation produces 116 million eggs, one million tons of beef worth USD 2.5 billion, and approximately 3.8 billion liters of milk valued at USD 5.1 billion [4].

The subsector has an enormous contribution to Ethiopia's national economy and the livelihoods of many Ethiopian farmers in the various farming systems and serves as a source of food, traction, manure, raw materials, cash income, and foreign exchange earnings and has social and cultural values [5]. The subsector contributes about 30% of the national gross domestic product (GDP) and 45% of the agricultural GDP [6]. It also contributes 15% of the export earnings [7]. However, the contribution of this huge livestock resource to the national income is not adequate and diseases are among the major factors responsible for poor production, productivity, and public health concerns [8]; hence, such enormous losses are mainly due to different diseases caused by bacteria, virus, and parasites [9].

Hydatid disease/echinococcosis is a parasitic disease caused by the larval stages of cestode belonging to the genus *Echinococcus*, which is one of the most common zoonotic diseases associated with huge economic losses and great public health significance [10, 11]. It is characterized by cysts containing numerous tiny protocolizes that most often develop in the liver and lungs and also in the kidneys, spleen, nervous tissue, bone, and other organs [12]. The parasites are perpetuated in life cycles with carnivore' definitive hosts, which harbor the adult with egg-producing stage in the intestine and intermediate host animals, in which the infective metacestode stage develops after oral infection with eggs [13]. The pathogenicity of hydatid disease heavily depends on the extent and severity of infection and the organ on which it is situated. The occasional rupture of hydatid cysts often leads to sudden death due to anaphylaxis, hemorrhage, and metastasis [14].

Hydatid diseases are associated with huge economic losses and great public health significance worldwide [11]. The disease is much more common in rural areas of Ethiopia where domestic animals live in a very close association with human beings as well as where home slaughtering of cattle, sheep, goats, and camels is still predominant and uncooked offal and carcass wastes are normally given to dogs and cats [15]. Several studies carried out in various regions of Ethiopia revealed that hydatid disease is extremely common and causes financial losses. Degefu and Damet [16] reported a USD 8792 annual direct loss from organ condemnation in Ethiopia.

Despite this disease's importance in Ethiopia, information on the current status of hydatid disease in cattle, and their implications on economic and public health concerns in different parts of the country particularly in Bishoftu Town are limited. The study of this health problem with the associated risk factors and determination of the economic significance are very important in planning and implementation of control strategies and to understand the risk of spreading of the disease both to domestic animals and humans.

Therefore, the objectives of this study were as follows.

### 1.1. Objectives

• To determine the prevalence of hydatid cysts and its association with the risk factors in cattle slaughtered at Bishoftu Town municipal abattoir,
• To evaluate economic loss of the hydatid disease, and
• To assess the community awareness about hydatid disease in the study area.

## 2. Materials and Methods

### 2.1. Study Area

The study was conducted from December 2022 to April 2023 at Bishoftu municipal abattoir, Oromia Region, Ethiopia. Bishoftu is located 47 km southeast of Addis Ababa, the capital city of the country. The altitude is about 1850 m above sea level. It experiences bimodal patterns of rainfall with the main rainy season extending from June to September with an average rainfall of about 800 mm. The mean annual minimum and maximum temperatures are 14°C and 26°C, respectively, with an overall average temperature of 20°C and the mean relative humidity is 61.3% [17]. Although the area has a few private abattoirs for ruminants with good facilities and precautions for protection against zoonotic disease, they are not yet providing service for local consumption. As a result, almost all people used either municipal abattoir or village slaughter with minor emphasis on meat quality and zoonotic disease concerns. There is only one municipal abattoir in the town which provides slaughtering services for an average of 50 heads of cattle per day.

### 2.2. Study Animals

The study animals were cattle brought to Bishoftu Town municipal abattoir from different areas for slaughtering purposes. A total of 480 cattle of both local and cross breeds of female and male animals with different body conditions and age groups were included in the study. The body condition score of animals was grouped as poor, medium, good, and obese [18] and their age was classified as < / = 6 years and > 6 years [19]. Animals were supplied to the abattoir from Bishoftu, Asella, Dukem, Modjo, Kaliti, Borena, and Adama, based on information from the suppliers. While most animals were sourced directly from breeders in these areas, some were also purchased from markets, making it difficult to accurately trace the origins of all the animals.

#### 2.2.1. Study Design

A cross-sectional study was conducted from December 2022 to April 2023 to study the prevalence of hydatid cysts in cattle at the Bishoftu municipal abattoir and to assess its direct and indirect financial losses. A questionnaire survey was used to assess the community's awareness of the public health importance of hydatid disease in cattle.

### 2.3. Sampling Methods and Sample Size Determination

The sample size was determined by the formula described by Thrusfield [20] with consideration of 50% expected prevalence considering there have not been any studies using a similar methodology in the field of interest for hydatidosis that this study addresses, with 5% desired absolute precision and 95% confidence level to calculate the minimal sample size.

(1) $$\begin{array}{c} n=\frac{1.96^{2}\times \text{Pexp}\left(1-\text{Pexp}\right)}{{\left(0.05\right)}^{2}}=1.962\times 0.5\left(1-0.5\right)=384, \end{array}$$

where *n* is the sample size, Pexp is the minimum expected prevalence (50%), 1.96 is the value of *Z* at 95% confidence interval, and *d* is the desired accuracy level at 95% confidence interval. The required sample size for this study was 384, but to increase precision, it was increased by 25–480%.

A questionnaire survey sample size was calculated based on the formula of Arsham [21].

*N* = 0.25/SE^2^, where *N* is the sample size and SE is the standard error assuming the standard error of 5% at a precision level of 0.05 and the confidence interval of 95%. 100 volunteer individuals were selected and interviewed with the consideration of different ages, sexes, religions, occupations, and educational levels.

Hydatid cysts were collected from different organs of slaughtered animals in the abattoir where they were selected systematically after every three animal intervals in a slaughterhouse.

### 2.4. Antemortem Examination of Cattle

1. In Bishoftu Town municipal abattoir, about 30–70 (on average 50) cattle were slaughtered per day. Both antemortem and postmortem examinations were carried out according to Herenda and Chambers, and Bekuma and his colleagues [22, 23].

As part of the antemortem examination, all animals were systematically assessed for signs of the disease, both while at rest and in motion within the lairage, to ensure that only apparently healthy animals were approved for slaughter. In addition, the animal's signalment information such as sex, age, origin, breed, and body condition was recorded before slaughtering on a specially prepared sheet.

### 2.5. Postmortem Examination of Cattle

In the abattoir, hydatid cysts were systematically collected from various organs of slaughtered animals, with sampling performed after every third animal. A postmortem examination was performed on the selected animals using visual inspection, palpation, and systematic incisions of the lungs, kidneys, liver, spleen, and heart to detect hydatid cysts [24, 25]. The number of cysts found in each organ was carefully recorded. Afterward, the cysts were properly labeled and transported to the veterinary parasitology and multipurpose laboratories at Addis Ababa University College of Veterinary Medicine and Agriculture (AAU-CVMA) for further characterization.

### 2.6. Hydatid Cysts Characterization

Hydatid cysts look like bladder-like pouches which are small around 5–10 cm in diameter and they are transparent and interspersed in the infected visceral organs [24]. The individual cysts from each of the infected organs of cattle were macroscopically examined for degeneration. After the hydatid cysts reached the laboratory, the cyst wall was penetrated by a needle and opened with scalpel blades. Then, the fluid contents were transferred into a sterile test tube. Based on the presence or absence of a broad capsule containing protocolizes in the hydatid fluid, cysts were classified as fertile or infertile. The cysts which contain protocolizes were classified as fertile cysts [26]. Fertile cysts were further subjected to viability tests to observe amoeboid-like peristaltic movement under a microscope objective of 40×. For clear vision, a drop of 0.1% aqueous eosin solution was added to cyst fluid on a microscope slide with the principle that viable protocolizes should completely or partially exclude the dye while the dead ones take it up [27]. The infertile cysts were further classified as sterile (fluid-filled cysts without any protocolizes) and calcified (if it is full of precipitation).

### 2.7. Economic Loss Estimation

Losses due to hydatid cyst–infected organs and tissue condemnation were calculated by considering information on the recent market price of the condemned organs and tissues [28] at butcher shops in Bishoftu. Direct losses were calculated based on the condemned organs and tissues and the indirect losses were estimated based on live weight losses caused by hydatid disease [29]. The total financial losses were calculated as the summation of the cost of offal's condemned plus the cost of carcass weight losses [30]. The average carcass weight of the Ethiopian local cattle breed is estimated as 126 kg [31], and eight different butcher men were asked randomly to determine the price per unit organ, and the average market price of one 1 kg of beef in Bishoftu Town was taken.

### 2.8. Community Awareness of Hydatid Disease

Partially organized questions focusing on knowledge of the community related to the public health concerns of hydatid disease and their practice, particularly toward the management of dogs which could get them vulnerable to this zoonotic disease were prepared and distributed to the respondents. These interviewees were included with consideration of their willingness and owning of cattle and their number was delaminated based on the formula [21].

### 2.9. Data Management and Analysis

All antemortem and postmortem findings from the abattoir and the data related to hydatid cyst characterizations in the laboratory were recorded and entered into an Excel sheet. The estimated direct and indirect financial losses are also recorded, calculated, and expressed in Ethiopian Birr (ETB). Descriptive statistics and statistical associations among variables were analyzed using STATA software @13. Questionnaire survey data were summarized using descriptive analysis. The finding was considered as 95% CI with the statistically significant level set at < 0.05.

## 3. Results

### 3.1. Overall Prevalence of Hydatid Cysts

Of the total 480 cattle examined, the overall prevalence of hydatid cysts in cattle slaughtered at the Bishoftu municipal abattoir was found to be 10.4% (50/480) (Table 1).

### 3.2. Prevalence Association With the Potential Risk Factors

From 193 animals under/at the age of 6 years, almost 17 (8.8%), and from 287 animals with the age above 6 years, about 33 (11.5%), were found to be harbor the hydatid cyst. There was no statistically significant difference (*p* > 0.05) between these two age groups in the prevalence of hydatid cysts. About 18 animals with poor, 274 medium, and 174 good body condition and 14 obese animals were examined and accounted for hydatid cysts presence of 4 (22.2%), 28 (10.2%), 18 (10.3%), and 0, respectively. There was no statistically significant variation (*p* > 0.05) in the prevalence of hydatid cysts among animals of these body conditions grouping (Table 1).

### 3.3. Burden and Distribution of Hydatid Cyst in Visceral Organ of Cattle

Out 480 cattle, 50 were found infected and 24 (5.0%) have hydatid cysts in their lungs, 13 (2.7%) in livers, 6 (1.3%) in the heart, 4 (0.8%) in kidneys, and 3 (0.6%) in the spleen represent the accounted shares from the total prevalence 10.4% (Table 2).

### 3.4. Hydatid Cyst Characterization Results

Those 85 hydatid cysts taken from different organs were subjected to laboratory tests and about 40 (47.1%) were fertile and 45 (52.9%) were infertile (Table 3). The cysts recovered from the lungs were more fertile than those from the liver, kidneys, spleen, and heart. The fertile cysts were subjected to a viability test indicating that 15/40 (37.5%) of the cysts were found to be viable and 25/40 (62.5%) were not viable (Table 4). In addition, infertile hydatid cysts were classified as sterile which were 17/45 (37.8%) and calcified were 28/45 (62.2%). In contrast, hydatid cysts from the heart were the most (100%) calcified cysts (Table 5).

### 3.5. Economical Loss Estimation Results of Hydatid Cyst

The annual direct financial loss due to cattle's visceral organ condemnation because of hydatid cyst infection was calculated by summation of the actual price of each organ in ETB at a time (Table 6). A total of about 255,380.40 ETB was estimated to be lost due to condemned lungs, liver, heart, spleen, and kidneys of cattle slaughtered in Bishoftu municipal abattoir per annum.

Indirect annual financial loss was calculated using the cost of carcass weight losses = 5% ∗ ACS ∗ PH ∗ AMP ∗ ACW.

  ACS = Average number of cattle slaughtered per year in Bishoftu municipal abattoir = 13,200.
  Five percent = Carcass weight losses in the individual animal due to hydatid disease
  PH = Prevalence of hydatid disease in Bishoftu municipal abattoir = 10.4%
  AMP = Average market price of 1 kg of beef in Bishoftu Town = 600 birrs.
  ACW = Average carcass weight of Ethiopian cattle = 126 kg.

As a result, the annual cost of carcass weight losses = 5% ∗ 13,200 ∗ 10.42% ∗ 600 ∗ 126 = 5,189,184.0 ETB. A total economic loss due to visceral organ condemnation and carcass weight losses was 255380.40 ETB + 5,189,184.0 ETB equals 5,444,564.4 ETB ($97,224.4), which is a very huge number indicating that hydatid disease is economically a very important disease in cattle in the study area.

### 3.6. Community Awareness Results on Hydatid Disease

Of the total 100 interviewed respondents of Bishoftu Town, 33% had no information about diseases transmitted from dogs to humans, and 62% did not know that dogs have involvement in hydatid cysts to be shared between humans and dogs. On the other hand, of 38 respondents who had awareness of the involvement of dogs in the transmission of CE to humans, 16 of them had no information about its control measures (Table 7).

Of the total respondents, 62% (62/100) had dogs that shared their house with them, and the potential predisposing factors associated with the hydatid cyst infection are dogs, attributed to the feeding of visceral organs of which 82.3% (51/62) dogs were found to be getting fed uncooked visceral organs (Table 8). Similarly, 51% of the respondents had no information on the importance of deworming animals in reducing the risk of the infection.

## 4. Discussion

Bovine hydatid disease is known to be an important disease in livestock and humans in different parts of the world and its prevalence and economic significance have been reported by different researchers in different geographical areas. The current study revealed that the overall prevalence of hydatid cysts in cattle slaughtered at Bishoftu municipal abattoir was 10.4%. This finding was comparable with previous study findings in Bako municipal abattoir reported (11.8%) by Berihu and Toffik [32], 12.2% in Elfora export abattoir reported by Worku [33], around 11.3% in Mizan Teppi municipal abattoir reported by Bekele et al. [34], 10.6% in Morocco reported by Berbri et al. [35], and 9.4% in Harar Town reported by Dinaol et al. [36]. However, it is lower than the previous findings of 52% in Southern Ethiopia reported by Guduro and Desta [37], 48.9% in Debre Markos by Kebede, Mitiku, and Tilahun [38], 63.7% in Asella municipal abattoir [39], and 33.6% in Mekelle municipal abattoir [40].

This variation in the prevalence of cystic echinococcosis among cattle of different areas could be attributed mainly to differences in agroecology, the times at which studies took place, stocking rates and movements of animals, animal husbandry systems, awareness, culture and religion of the society, and attitude towards dogs' management in different regions [41, 42]. It also could be due to the variation in *Echinococcus granulosus* strains that exist in different geographical locations [43].

In this study, the lungs were found to be the most commonly affected organ (48%) by hydatid cysts followed by the liver (26%). This will be explained by the fact that the lung and liver have the first large capillary sites that the migrating *Echinococcus oncosphere* encounters as it follows the portal vein route before it encounters any other peripheral organs. This finding agreed with the finding of [44], which reported 50.5% in the lungs and 40.6% in the liver and the findings of [45], which reported 54.5% in the lungs and 43.5% in the liver. The softer nature of the lungs is the cause of the increased percentage of cysts there. The fact that most cattle are slaughtered at older ages may be the second explanation. The liver capillaries dilate during this time, and the majority of cysts travel straight to the lungs. The hexacanth embryo may also enter the lymphatic system and go to the heart, lungs, and liver through the thoracic duct, infecting the lungs before the liver. However, this study found a higher prevalence of hydatid cysts than the findings of [46], which reported 12.5% and 4.3% in the lungs and liver, respectively.

The body condition categories of animals have not shown significant statistical (*p* > 0.05) variation with the prevalence of hydatid cysts. The reason could be that the primary difference in body condition might be caused by several other additional factors and might be due to the little tendency to exclude emaciated animals from being slaughtered. Contrary to this study's finding, the authors in [47, 48] reported a higher prevalence of hydatid cysts in animals with poor body condition than in the respective medium and good counterparts.

Similarly, there is no statistically significant association between the age category of animals. All these risk factors have no statistically significant association (*p* > 0.05) with the prevalence of hydatid cyst infection in the study area. The possible reason might be that the animals have managed in a similar way creating relatively comparable spatial and temporal exposure. However, a higher prevalence of hydatid cysts in older animals was reported by the authors in [47, 49].

Fertility of hydatid cysts in various organs of cattle is an important indicator of potential sources of infection to perpetuate the disease in dogs. In this study, hydatid cysts recovered from different organs showed variation in fertility rate although not statistically significantly associated. Lungs took the higher proportion at 59.6% of the fertility rate followed by the liver at 40% than the rest of the visceral organs. This showed agreement with earlier findings of [50] that reported 32.7% in the lungs and 32.6% in the liver and the findings of [24] that reported 55.2% in the lungs and 37.1% in the liver but it disagrees with the finding of [51], which reported 3.1% in the lungs and 5.2% in the liver. This variation might be due to the difference in the infected host and the difference in tissue resistance of organs. The percentage of calcified cysts is observed to be higher in the liver than in the lungs. The result agreed with the study of [47]. This might be related to the liver being firm in consistency and lacking a suitable matrix for long-term cyst survival and hence the cyst degenerates earlier than the one found in the lungs [52]. It could also be attributed to the organ's substantial connective tissue reactivity and significantly higher reticuloendothelial cell density [53].

The current total financial annual loss of 5,444,564.4 ETB ($97,224.4) estimated financial losses from bovine hydatid disease. This finding was much higher than $21,833.60 reported in Dire Dawa [54], 8561.61 ETB ($450.6) reported in Nekemte [55], and 96,315.00 ETB ($4815.7) reported in Harar [56]. These variations could arise from organ price variation among study sites and price inflation of the organ which is constantly increasing from year to year. The figure for the financial loss due to organ condemnation in the present study is significant for the country, i.e., endeavoring to thrive from economic problems.

In Bishoftu Town, the community awareness regarding the public health importance and preventive measures against such important neglected zoonosis and risky practices that could contribute to the spread and persistence of the disease was still lower. In this study, 61% of the respondents did not know CE disease, 70% of the respondents were not aware that CE is dangerous to human health, and 62% of them were not aware of how a human can acquire the disease. Compared to the present study's finding, a study in Morocco concluded an even lower level of awareness regarding CE with only 20% of the interviewees realizing that dogs play a role in the transmission of CE in humans and animals [57].

Several potential risky practices have been underlined among the respondents interviewed in this study, notably practices related to dog management, and about 40.3% of the respondents complained that they never tie up their dogs. This finding reflects a poor awareness among the respondents regarding the role of dogs in cystic echinococcosis transmission. About 51.6% of the respondents did not deworm their dogs, and 82.3% of respondents were feeding uncooked viscera organs to their dogs. Such risky practices have been among the most important factors that increase contamination of the environment with faces containing echinococcosis eggs [58]. Similar findings have been documented in other cystic echinococcosis-endemic countries. A study in Sardinia (Italy) found that the majority of the interviewed farmers used raw offal, after home slaughtering, to feed their dogs [59]. Moreover, a study in Tibet demonstrated that feeding dogs with uncooked viscera is a risk factor for increasing the likelihood of human infection with *E. granulosus* [60].

## 5. Conclusion and Recommendations

Hydatid disease is a zoonotic disease that causes cyst formation in internal tissues such as the liver, lungs, and brain of grazing animals such as cattle, and also people. Although the findings of this study showed a relatively lower prevalence of hydatid cysts, it is still a potential health problem for both cattle and humans in Bishoftu Town. More importantly, the majority of the community in this study was at risk of contracting the disease due to a lack of knowledge on transmission, zoonosis, and control of hydatid cysts. It also relayed a significant economic loss either via direct or indirect means in cattle, with a high public health threat in the study area. Although the disease has animal and human health accusation and is economically hurtful, it did not get praiseworthy attention from the stockholders and it continues to impose its impact on animals and humans.

Therefore, based on this conclusion, the following recommendations are forwarded:

• The dog population should be properly controlled and provided strategic deworming to halt the source and distribution of cystic echinococcosis infection to the intermediate hosts and humans in the study area.
• Advanced studies with considerations variety in the strains of *E. granulosus* both in the final and intermediate hosts should be conducted to tackle the problem in Bishoftu Town and at the national level at large.

## Figures and Tables

**Table 1:** Prevalence of hydatid cyst with the associated risk factors of cattle slaughtered at Bishoftu municipal abattoir (*N* = 480).

Risk factors	Category	Examined animals	Positive animals	Prevalence (%)	*X* ^2^	*p* value
Sex	Female	24	3	12.5	0.12	0.73
	Male	456	47	10.3		

Age	< / = 6 years	193	17	8.8	0.90	0.34
	> 6 years	287	33	11.5		

	Local	465	48	10.3	0.14	0.707
	Cross	15	2	13.3		

BCS	Poor	18	4	22.2	4.33	0.23
	Medium	274	28	10.2		
	Good	174	18	10.3		
	Obese	14	0	0		

Overall		480	50	10.4		

**Table 2:** Proportional distribution of hydatid cyst infection in visceral organs of cattle.

Organs	Hydatid cyst–infected organs (*n* = 50)	Hydatid cysts–infected animals (*n* = 480) (%)
Lungs	24 (48%)	5.0
Liver	13 (26%)	2.7
Heart	6 (12%)	1.3
Spleen	3 (6%)	0.6
Kidneys	4 (8%)	0.8
Total	50 (100%)	10.4

**Table 3:** Fertility of hydatid cyst per visceral organ of recovery in cattle using logistic regression (STATA 13@).

Organ of recovery	Recovered cyst	Fertile cyst	OR value	95% CI	*X* ^2^	*p* value
Spleen	3	1	—	—	—	—
Kidneys	4	1	0.667	0.025–18.056	−0.24	0.810
Heart	6	0	1	—	—	—
Liver	25	10	1.333	0.106–16.743	0.22	0.824
Lungs	47	28	2.947	0.249–34.850	0.86	0.391
Overall (%)	85/480	45/85	0.5	0.0453–5.514	−0.57	0.571

**Table 4:** Viability of fertile hydatid cysts' association with an organ of recovery in cattle (*N* = 40) (STATA13@).

Total fertile cyst per organ of recovery	Viable cyst (%)	Nonviable (%)	*X* ^2^	*p* value
Spleen (1)	1	0	3.2762	0.351
Kidneys (1)	0	1		
Heart (0)	—	—		
Liver (10)	5	5		
Lungs (28)	9	19		
Overall (40)	15/40	25/40		

**Table 5:** Calcification and sterility of infertile hydatid cysts' association with an organ of recovery in cattle using the chi-square test (STATA 13@).

Infertile cyst per organ of recovery	Calcified cysts (%)	Sterile cysts (%)	*X* ^2^	*p* value
Spleen (2)	2	0	8.2795	0.082
Kidneys 3)	2	1		
Heart (6)	6	0		
Liver (15)	10	5		
Lungs 19)	8	11		
Overall (45)	28/45	17/45		

**Table 6:** Estimation of financial loss due to condemnation of hydatid cyst-infected organs in cattle.

Organs	No. of organs condemned	% of condemned	Average price per organ	Annual total price in ETB
Lungs	24	5	30	19800.00
Liver	13	2.70	550	196020.00
Heart	6	1.25	150	24750.00
Spleen	3	0. 63	20	1663.20
Kidneys	4	0.83	120	13147.20
Total	50	10.41	870	255380.40

**Table 7:** Community awareness on public health importance of hydatid disease in cattle in Bishoftu Town on 100 respondents owning cattle and dogs.

Question's themes	Respondents	Yes	No
Do you know diseases are transmitted from dogs to human?	100	67	33
Do you know about echinococcosis disease?	100	39	61
Do you know CE can be dangerous to human health?	100	30	70
Do you know dogs are involved in the transmission of CE to humans?	100	38	62
Do you know the control methods for hydatid disease infection?	38	22	16

**Table 8:** Cystic echinococcosis–related managemental practice in dogs by the community in Bishoftu Town.

Question's themes	Respondents	Yes	No
Do you share your house with dogs?	62	62	0
Do you tie up your dog?	62	37	25
Do you feed uncooked visceral organs of cattle to your dog?	62	51	11
Do you get deworming for your dogs?	62	30	32

## Data Availability

The data used to support the findings of this study are available from the corresponding author upon request.
